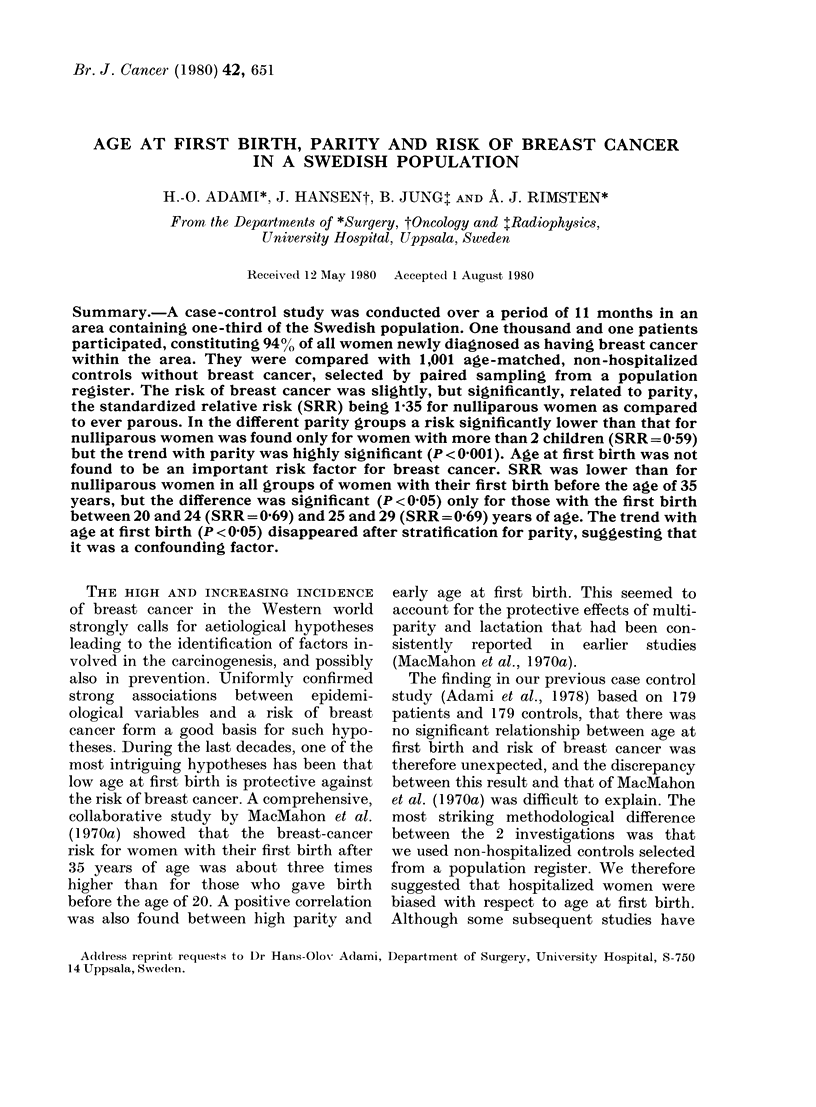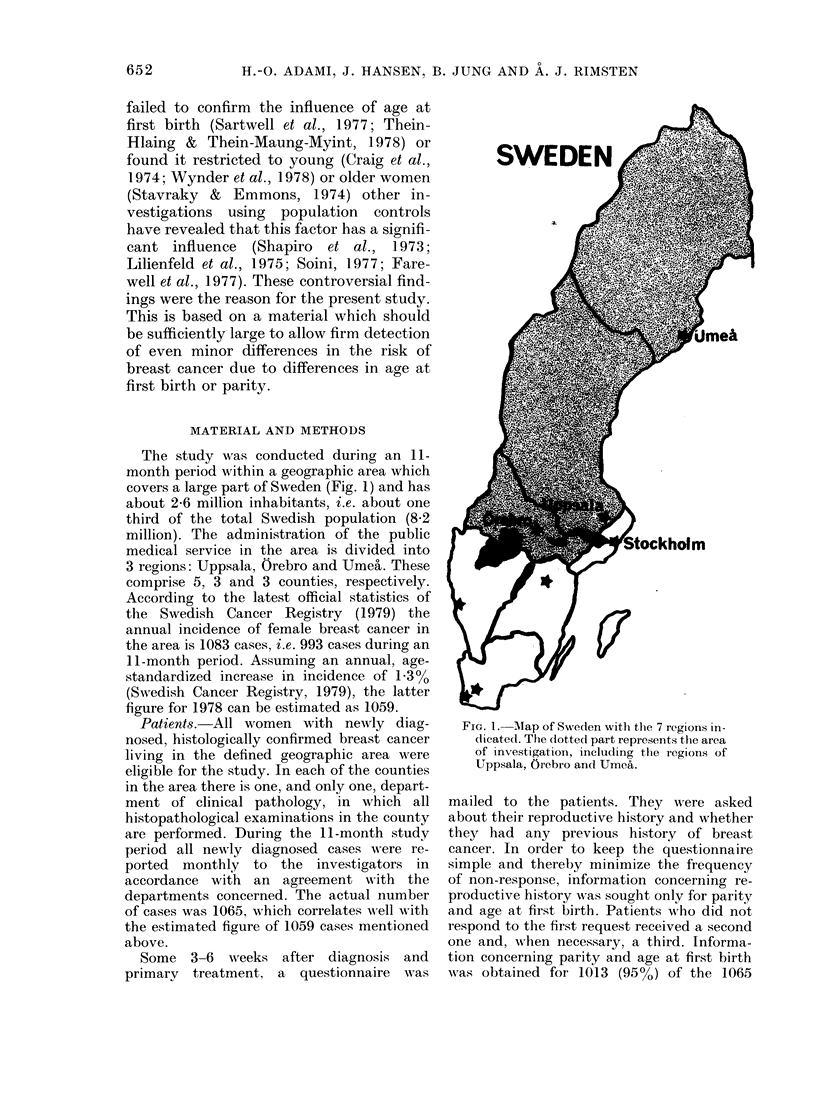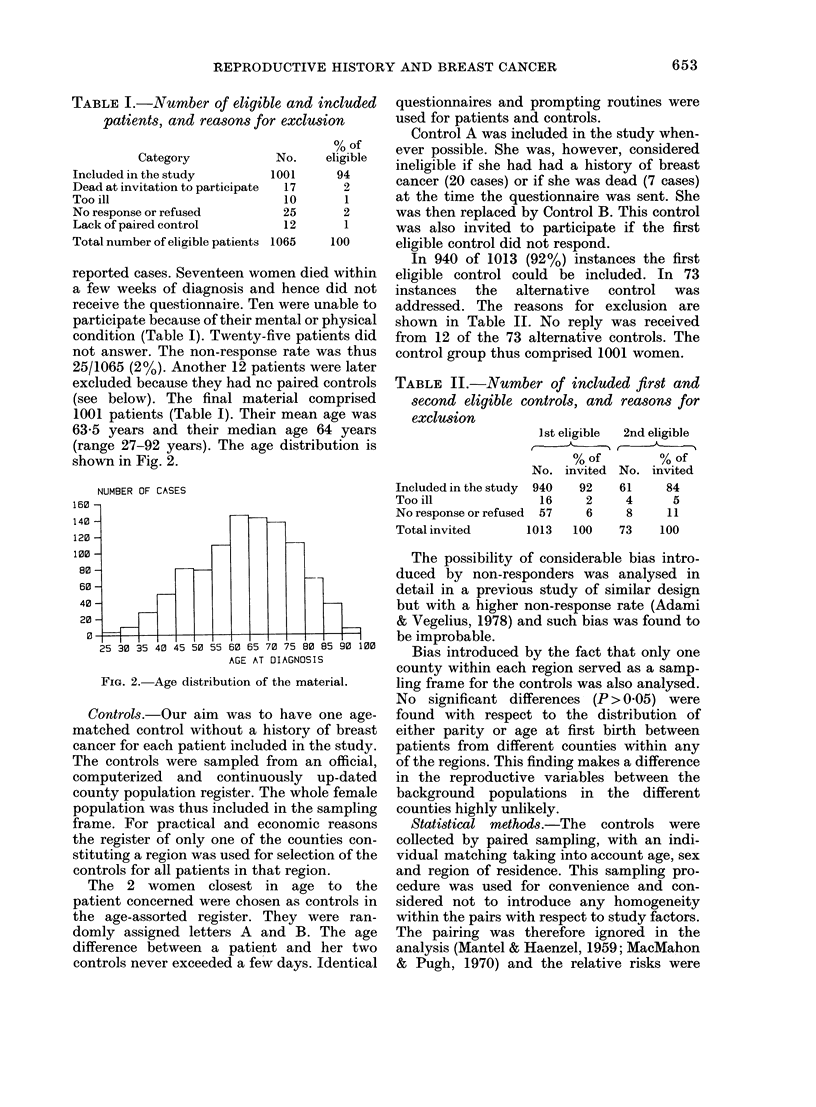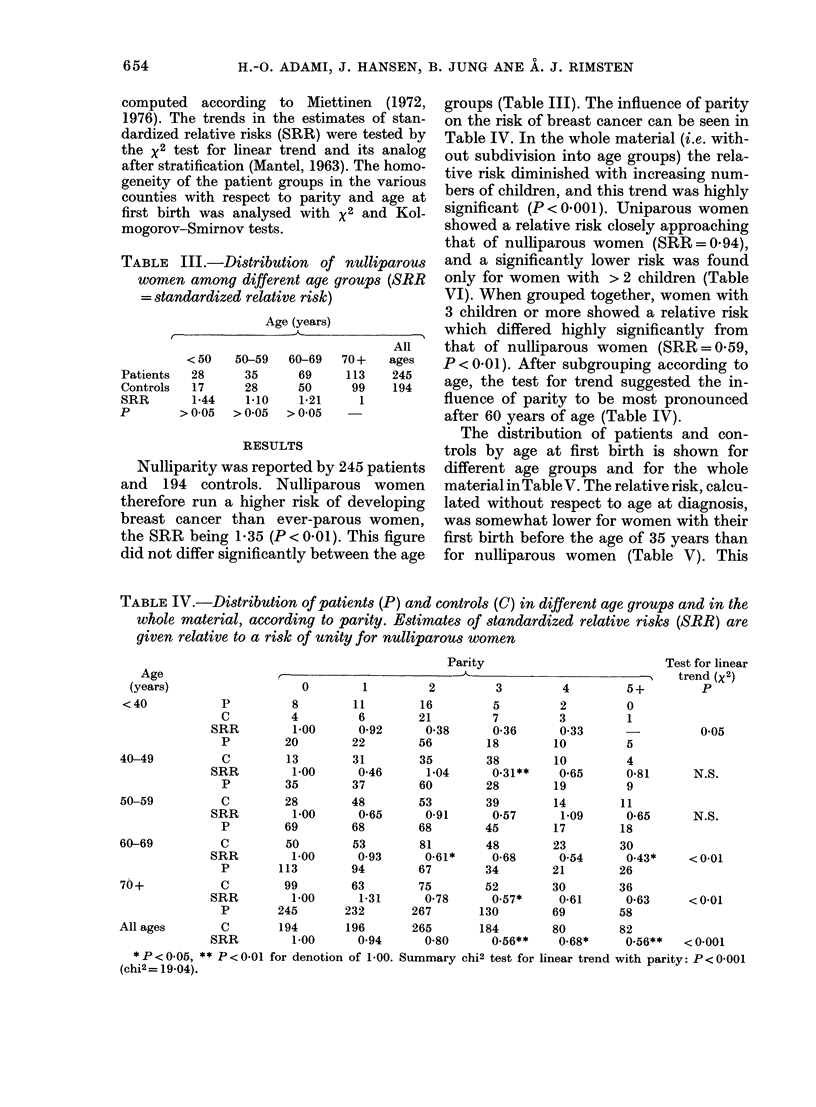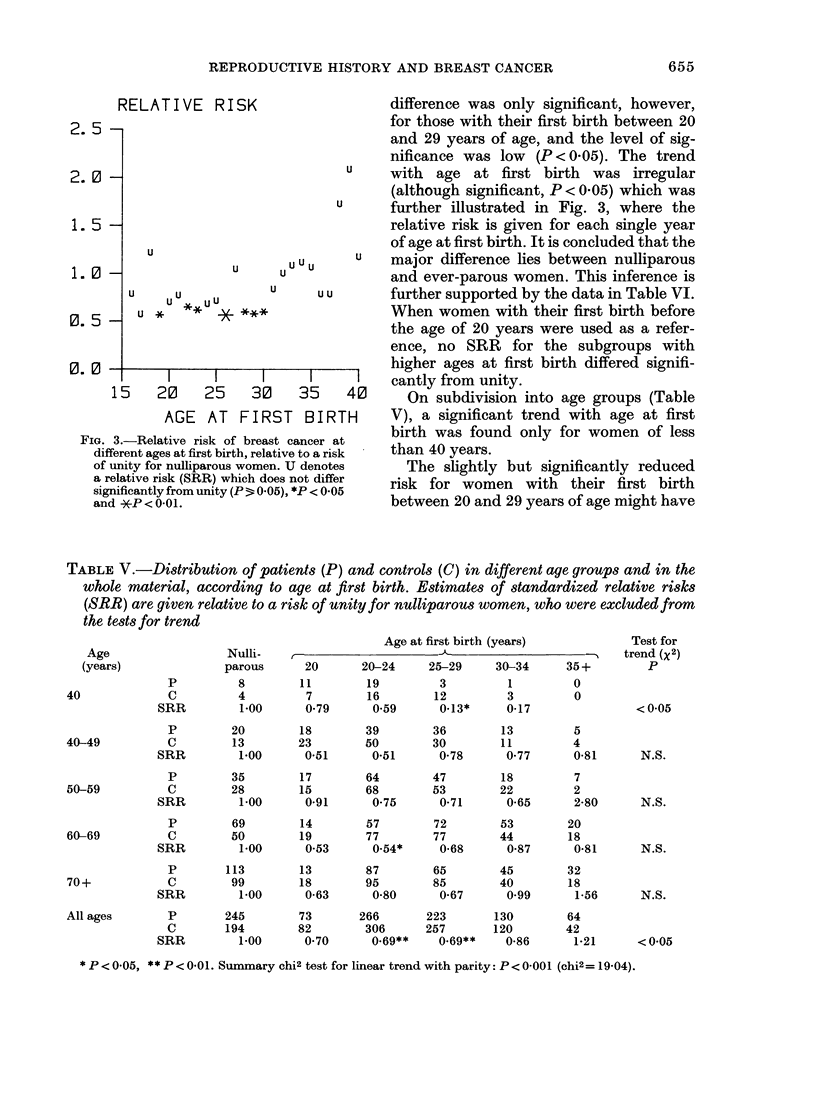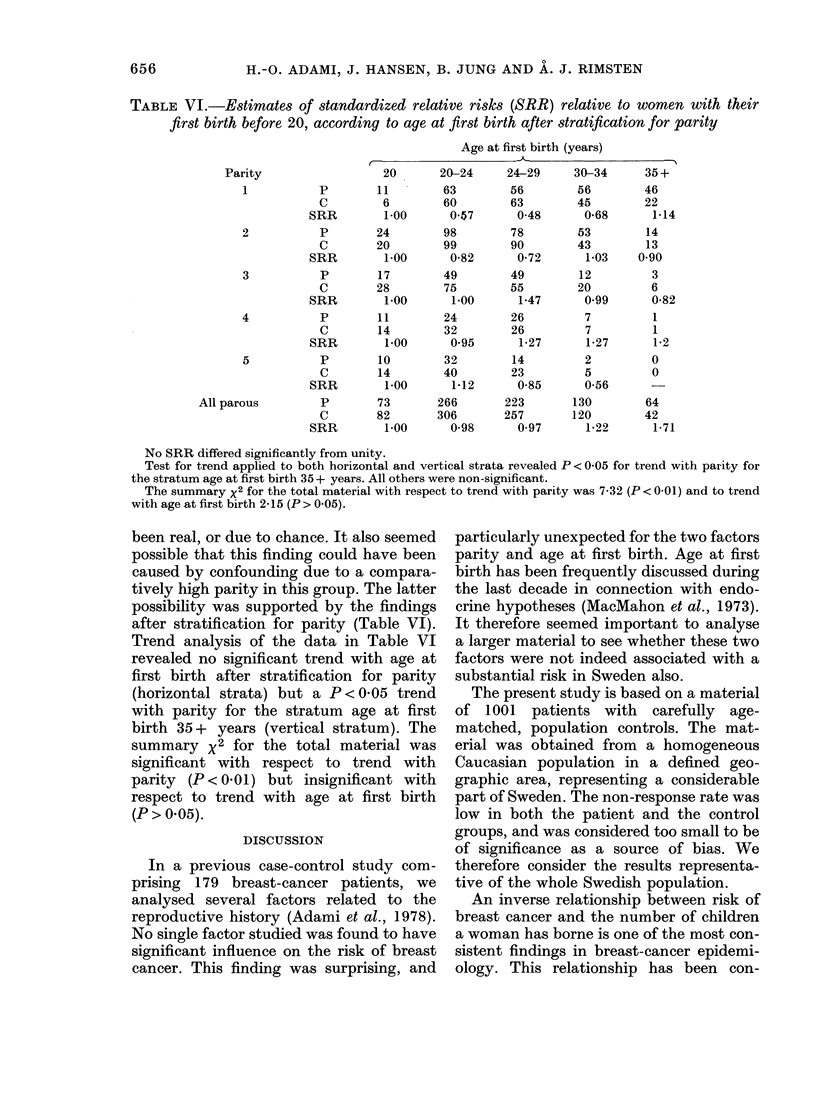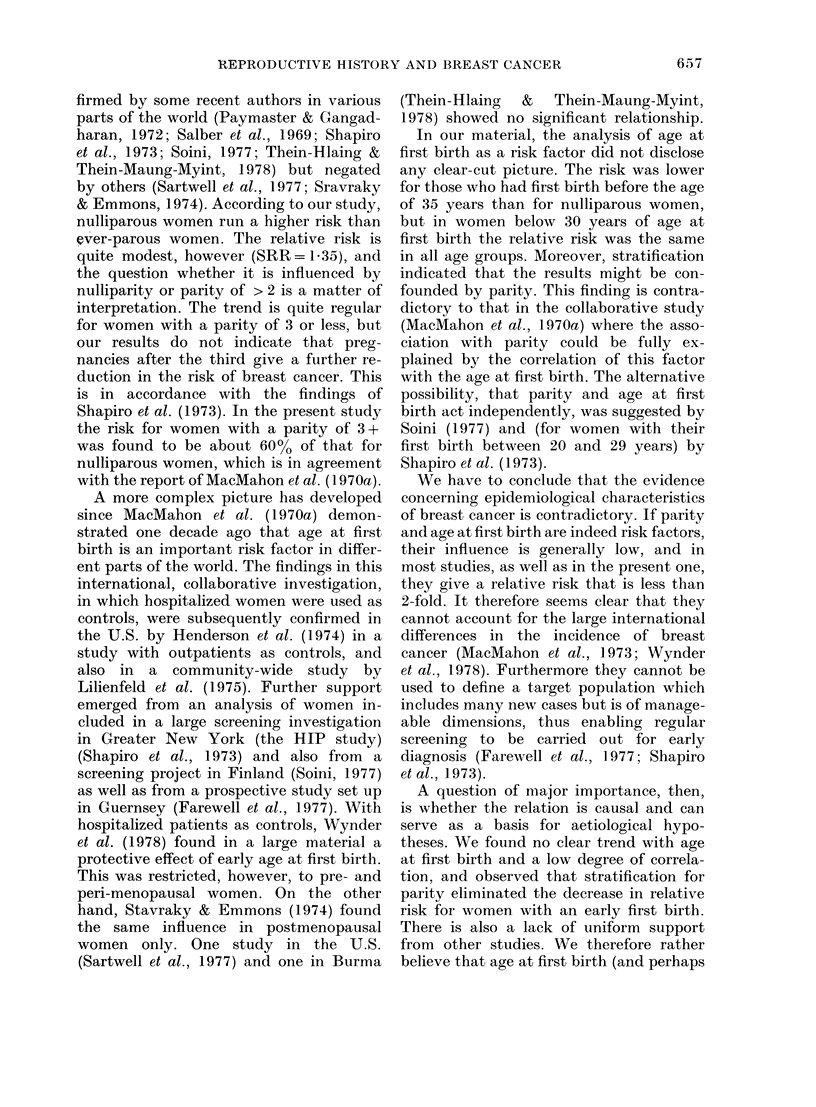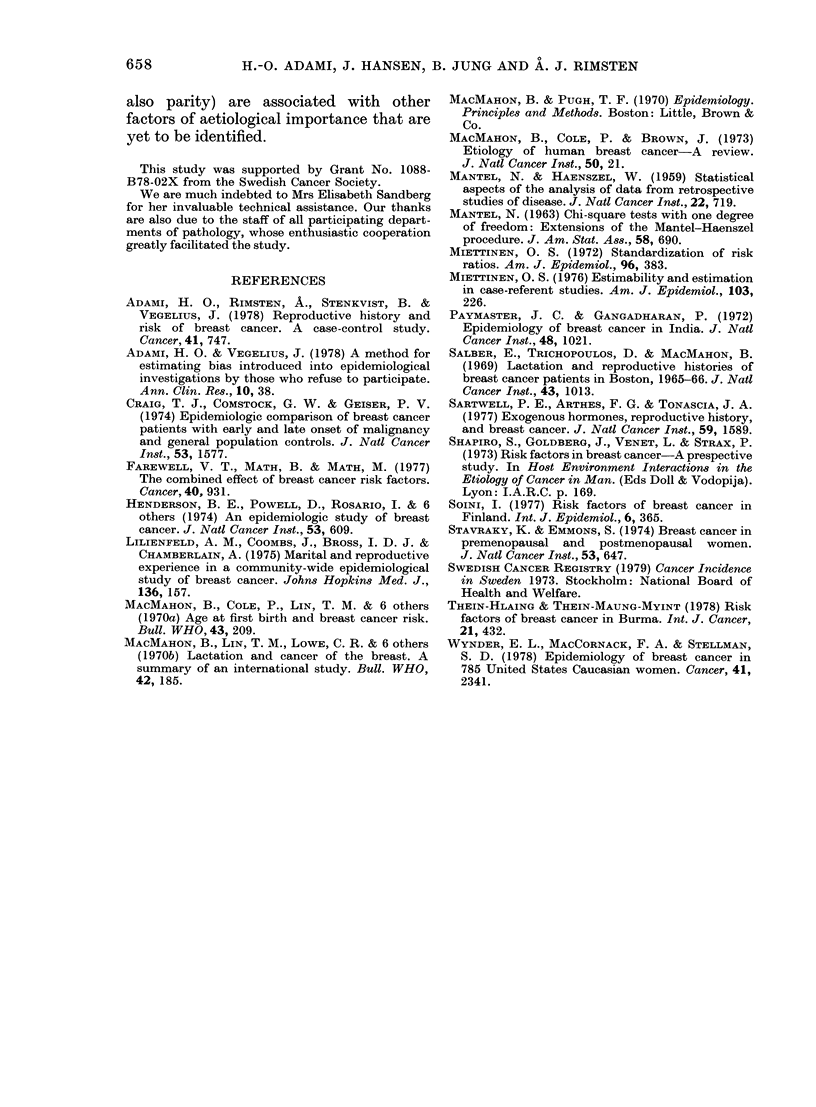# Age at first birth, parity and risk of breast cancer in a Swedish population.

**DOI:** 10.1038/bjc.1980.298

**Published:** 1980-11

**Authors:** H. O. Adami, J. Hansen, B. Jung, A. J. Rimsten

## Abstract

A case-control study was conducted over a period of 11 months in an area containing one-third of the Swedish population. One thousand and one patients participated, constituting 94% of all women newly diagnosed as having breast cancer within the area. They were compared with 1,001 age-matched, non-hospitalized controls without breast cancer, selected by paired sampling from a population register. The risk of breast cancer was slightly, but significantly, related to parity, the standardized relative risk (SRR) being 1.35 for nulliparous women as compared to ever parous. In the different parity groups a risk significantly lower than that for nulliparous women was found only for women with more than 2 children (SRR = 0.59) but the trend with parity was highly significant (P less than 0.001). Age at first birth was not found to be an important risk factor for breast cancer. SRR was lower than for nulliparous women in all groups of women with their first birth before the age of 35 years, but the difference was significant (P less than 0.05) only for those with the first birth between 20 and 24 (SSR = 0.69) and 25 and 29 (SRR = 0.69) years of age. The trend with age at first birth (P less than 0.05) disappeared after stratification for parity, suggesting that it was a confounding factor.


*Br. J. Cancer* (1980) **42**, 651

### AGE AT FIRST BIRTH, PARITY AND RISK OF BREAST CANCER IN A SWEDISH POPULATION

H.-O. ADAMI*, J. HANSEN†, B. JUNG‡ AND Å. J. RIMSTEN*

*From the Departments of *Surgery, †Oncology and ‡Radiophysics, University Hospital, Uppsala, Sweden*

Received 12 May 1980 Accepted 1 August 1980

**Summary.—A case-control study was conducted over a period of 11 months in an area containing one-third of the Swedish population. One thousand and one patients participated, constituting 94% of all women newly diagnosed as having breast cancer within the area. They were compared with 1,001 age-matched, non-hospitalized controls without breast cancer, selected by paired sampling from a population register. The risk of breast cancer was slightly, but significantly, related to parity, the standardized relative risk (SRR) being 1·35 for nulliparous women as compared to ever parous. In the different parity groups a risk significantly lower than that for nulliparous women was found only for women with more than 2 children (SRR = 0·59) but the trend with parity was highly significant ($P < 0·001$). Age at first birth was not found to be an important risk factor for breast cancer. SRR was lower than for nulliparous women in all groups of women with their first birth before the age of 35 years, but the difference was significant ($P < 0·05$) only for those with the first birth between 20 and 24 (SRR = 0·69) and 25 and 29 (SRR = 0·69) years of age. The trend with age at first birth ($P < 0·05$) disappeared after stratification for parity, suggesting that it was a confounding factor.**

THE HIGH AND INCREASING INCIDENCE of breast cancer in the Western world strongly calls for aetiological hypotheses leading to the identification of factors involved in the carcinogenesis, and possibly also in prevention. Uniformly confirmed strong associations between epidemiological variables and a risk of breast cancer form a good basis for such hypotheses. During the last decades, one of the most intriguing hypotheses has been that low age at first birth is protective against the risk of breast cancer. A comprehensive, collaborative study by MacMahon *et al.* (1970*a*) showed that the breast-cancer risk for women with their first birth after 35 years of age was about three times higher than for those who gave birth before the age of 20. A positive correlation was also found between high parity and early age at first birth. This seemed to account for the protective effects of multiparity and lactation that had been consistently reported in earlier studies (MacMahon *et al.*, 1970*a*).

The finding in our previous case control study (Adami *et al.*, 1978) based on 179 patients and 179 controls, that there was no significant relationship between age at first birth and risk of breast cancer was therefore unexpected, and the discrepancy between this result and that of MacMahon *et al.* (1970*a*) was difficult to explain. The most striking methodological difference between the 2 investigations was that we used non-hospitalized controls selected from a population register. We therefore suggested that hospitalized women were biased with respect to age at first birth. Although some subsequent studies have

Address reprint requests to Dr Hans-Olov Adami, Department of Surgery, University Hospital, S-750 14 Uppsala, Sweden.

failed to confirm the influence of age at first birth (Sartwell *et al.*, 1977; Thein-Hlaing & Thein-Maung-Myint, 1978) or found it restricted to young (Craig *et al.*, 1974; Wynder *et al.*, 1978) or older women (Stavraky & Emmons, 1974) other investigations using population controls have revealed that this factor has a significant influence (Shapiro *et al.*, 1973; Lilienfeld *et al.*, 1975; Soini, 1977; Farewell *et al.*, 1977). These controversial findings were the reason for the present study. This is based on a material which should be sufficiently large to allow firm detection of even minor differences in the risk of breast cancer due to differences in age at first birth or parity.

FIG. 1.—Map of Sweden with the 7 regions indicated. The dotted part represents the area of investigation, including the regions of Uppsala, Örebro and Umeå.

### MATERIAL AND METHODS

The study was conducted during an 11-month period within a geographic area which covers a large part of Sweden (Fig. 1) and has about 2·6 million inhabitants, *i.e.* about one third of the total Swedish population (8·2 million). The administration of the public medical service in the area is divided into 3 regions: Uppsala, Örebro and Umeå. These comprise 5, 3 and 3 counties, respectively. According to the latest official statistics of the Swedish Cancer Registry (1979) the annual incidence of female breast cancer in the area is 1083 cases, *i.e.* 993 cases during an 11-month period. Assuming an annual, age-standardized increase in incidence of 1·3% (Swedish Cancer Registry, 1979), the latter figure for 1978 can be estimated as 1059.

*Patients.*—All women with newly diagnosed, histologically confirmed breast cancer living in the defined geographic area were eligible for the study. In each of the counties in the area there is one, and only one, department of clinical pathology, in which all histopathological examinations in the county are performed. During the 11-month study period all newly diagnosed cases were reported monthly to the investigators in accordance with an agreement with the departments concerned. The actual number of cases was 1065, which correlates well with the estimated figure of 1059 cases mentioned above.

Some 3–6 weeks after diagnosis and primary treatment, a questionnaire was mailed to the patients. They were asked about their reproductive history and whether they had any previous history of breast cancer. In order to keep the questionnaire simple and thereby minimize the frequency of non-response, information concerning reproductive history was sought only for parity and age at first birth. Patients who did not respond to the first request received a second one and, when necessary, a third. Information concerning parity and age at first birth was obtained for 1013 (95%) of the 1065

TABLE I.—*Number of eligible and included patients, and reasons for exclusion*

| Category | No. | % of eligible |
|---|---|---|
| Included in the study | 1001 | 94 |
| Dead at invitation to participate | 17 | 2 |
| Too ill | 10 | 1 |
| No response or refused | 25 | 2 |
| Lack of paired control | 12 | 1 |
| Total number of eligible patients | 1065 | 100 |

reported cases. Seventeen women died within a few weeks of diagnosis and hence did not receive the questionnaire. Ten were unable to participate because of their mental or physical condition (Table I). Twenty-five patients did not answer. The non-response rate was thus 25/1065 (2%). Another 12 patients were later excluded because they had nc paired controls (see below). The final material comprised 1001 patients (Table I). Their mean age was 63·5 years and their median age 64 years (range 27–92 years). The age distribution is shown in Fig. 2.

FIG. 2.—Age distribution of the material.

*Controls.*—Our aim was to have one age-matched control without a history of breast cancer for each patient included in the study. The controls were sampled from an official, computerized and continuously up-dated county population register. The whole female population was thus included in the sampling frame. For practical and economic reasons the register of only one of the counties constituting a region was used for selection of the controls for all patients in that region.

The 2 women closest in age to the patient concerned were chosen as controls in the age-assorted register. They were randomly assigned letters A and B. The age difference between a patient and her two controls never exceeded a few days. Identical questionnaires and prompting routines were used for patients and controls.

Control A was included in the study whenever possible. She was, however, considered ineligible if she had had a history of breast cancer (20 cases) or if she was dead (7 cases) at the time the questionnaire was sent. She was then replaced by Control B. This control was also invited to participate if the first eligible control did not respond.

In 940 of 1013 (92%) instances the first eligible control could be included. In 73 instances the alternative control was addressed. The reasons for exclusion are shown in Table II. No reply was received from 12 of the 73 alternative controls. The control group thus comprised 1001 women.

TABLE II.—*Number of included first and second eligible controls, and reasons for exclusion*

| | 1st eligible | | 2nd eligible | |
|---|---|---|---|---|
| | No. | % of invited | No. | % of invited |
| Included in the study | 940 | 92 | 61 | 84 |
| Too ill | 16 | 2 | 4 | 5 |
| No response or refused | 57 | 6 | 8 | 11 |
| Total invited | 1013 | 100 | 73 | 100 |

The possibility of considerable bias introduced by non-responders was analysed in detail in a previous study of similar design but with a higher non-response rate (Adami & Vegelius, 1978) and such bias was found to be improbable.

Bias introduced by the fact that only one county within each region served as a sampling frame for the controls was also analysed. No significant differences ($P > 0{\cdot}05$) were found with respect to the distribution of either parity or age at first birth between patients from different counties within any of the regions. This finding makes a difference in the reproductive variables between the background populations in the different counties highly unlikely.

*Statistical methods.*—The controls were collected by paired sampling, with an individual matching taking into account age, sex and region of residence. This sampling procedure was used for convenience and considered not to introduce any homogeneity within the pairs with respect to study factors. The pairing was therefore ignored in the analysis (Mantel & Haenzel, 1959; MacMahon & Pugh, 1970) and the relative risks were computed according to Miettinen (1972, 1976). The trends in the estimates of standardized relative risks (SRR) were tested by the $\chi^2$ test for linear trend and its analog after stratification (Mantel, 1963). The homogeneity of the patient groups in the various counties with respect to parity and age at first birth was analysed with $\chi^2$ and Kolmogorov–Smirnov tests.

TABLE III.—*Distribution of nulliparous women among different age groups (SRR = standardized relative risk)*

| | Age (years) | | | | |
|---|---|---|---|---|---|
| | < 50 | 50–59 | 60–69 | 70 + | All ages |
| Patients | 28 | 35 | 69 | 113 | 245 |
| Controls | 17 | 28 | 50 | 99 | 194 |
| SRR | 1·44 | 1·10 | 1·21 | 1 | |
| *P* | > 0·05 | > 0·05 | > 0·05 | — | |

### RESULTS

Nulliparity was reported by 245 patients and 194 controls. Nulliparous women therefore run a higher risk of developing breast cancer than ever-parous women, the SRR being 1·35 ($P < 0{\cdot}01$). This figure did not differ significantly between the age groups (Table III). The influence of parity on the risk of breast cancer can be seen in Table IV. In the whole material (*i.e.* without subdivision into age groups) the relative risk diminished with increasing numbers of children, and this trend was highly significant ($P < 0{\cdot}001$). Uniparous women showed a relative risk closely approaching that of nulliparous women (SRR = 0·94), and a significantly lower risk was found only for women with > 2 children (Table VI). When grouped together, women with 3 children or more showed a relative risk which differed highly significantly from that of nulliparous women (SRR = 0·59, $P < 0{\cdot}01$). After subgrouping according to age, the test for trend suggested the influence of parity to be most pronounced after 60 years of age (Table IV).

The distribution of patients and controls by age at first birth is shown for different age groups and for the whole material in Table V. The relative risk, calculated without respect to age at diagnosis, was somewhat lower for women with their first birth before the age of 35 years than for nulliparous women (Table V). This

TABLE IV.—*Distribution of patients (P) and controls (C) in different age groups and in the whole material, according to parity. Estimates of standardized relative risks (SRR) are given relative to a risk of unity for nulliparous women*

| Age (years) | | Parity 0 | 1 | 2 | 3 | 4 | 5+ | Test for linear trend ($\chi^2$) *P* |
|---|---|---|---|---|---|---|---|---|
| < 40 | P | 8 | 11 | 16 | 5 | 2 | 0 | |
| | C | 4 | 6 | 21 | 7 | 3 | 1 | |
| | SRR | 1·00 | 0·92 | 0·38 | 0·36 | 0·33 | — | 0·05 |
| | P | 20 | 22 | 56 | 18 | 10 | 5 | |
| 40–49 | C | 13 | 31 | 35 | 38 | 10 | 4 | |
| | SRR | 1·00 | 0·46 | 1·04 | 0·31** | 0·65 | 0·81 | N.S. |
| | P | 35 | 37 | 60 | 28 | 19 | 9 | |
| 50–59 | C | 28 | 48 | 53 | 39 | 14 | 11 | |
| | SRR | 1·00 | 0·65 | 0·91 | 0·57 | 1·09 | 0·65 | N.S. |
| | P | 69 | 68 | 68 | 45 | 17 | 18 | |
| 60–69 | C | 50 | 53 | 81 | 48 | 23 | 30 | |
| | SRR | 1·00 | 0·93 | 0·61* | 0·68 | 0·54 | 0·43* | < 0·01 |
| | P | 113 | 94 | 67 | 34 | 21 | 26 | |
| 70+ | C | 99 | 63 | 75 | 52 | 30 | 36 | |
| | SRR | 1·00 | 1·31 | 0·78 | 0·57* | 0·61 | 0·63 | < 0·01 |
| | P | 245 | 232 | 267 | 130 | 69 | 58 | |
| All ages | C | 194 | 196 | 265 | 184 | 80 | 82 | |
| | SRR | 1·00 | 0·94 | 0·80 | 0·56** | 0·68* | 0·56** | < 0·001 |

* $P < 0{\cdot}05$, ** $P < 0{\cdot}01$ for denotion of 1·00. Summary $chi^2$ test for linear trend with parity: $P < 0{\cdot}001$ ($chi^2 = 19{\cdot}04$).

FIG. 3.—Relative risk of breast cancer at different ages at first birth, relative to a risk of unity for nulliparous women. U denotes a relative risk (SRR) which does not differ significantly from unity ($P \geqslant 0{\cdot}05$), *$P < 0{\cdot}05$ and ✱$P < 0{\cdot}01$.

difference was only significant, however, for those with their first birth between 20 and 29 years of age, and the level of significance was low ($P < 0{\cdot}05$). The trend with age at first birth was irregular (although significant, $P < 0{\cdot}05$) which was further illustrated in Fig. 3, where the relative risk is given for each single year of age at first birth. It is concluded that the major difference lies between nulliparous and ever-parous women. This inference is further supported by the data in Table VI. When women with their first birth before the age of 20 years were used as a reference, no SRR for the subgroups with higher ages at first birth differed significantly from unity.

On subdivision into age groups (Table V), a significant trend with age at first birth was found only for women of less than 40 years.

The slightly but significantly reduced risk for women with their first birth between 20 and 29 years of age might have

TABLE V.—*Distribution of patients (P) and controls (C) in different age groups and in the whole material, according to age at first birth. Estimates of standardized relative risks (SRR) are given relative to a risk of unity for nulliparous women, who were excluded from the tests for trend*

| Age (years) | | Nulliparous | Age at first birth (years): 20 | 20–24 | 25–29 | 30–34 | 35+ | Test for trend ($\chi^2$) *P* |
|---|---|---|---|---|---|---|---|---|
| | P | 8 | 11 | 19 | 3 | 1 | 0 | |
| 40 | C | 4 | 7 | 16 | 12 | 3 | 0 | |
| | SRR | 1·00 | 0·79 | 0·59 | 0·13* | 0·17 | | < 0·05 |
| | P | 20 | 18 | 39 | 36 | 13 | 5 | |
| 40–49 | C | 13 | 23 | 50 | 30 | 11 | 4 | |
| | SRR | 1·00 | 0·51 | 0·51 | 0·78 | 0·77 | 0·81 | N.S. |
| | P | 35 | 17 | 64 | 47 | 18 | 7 | |
| 50–59 | C | 28 | 15 | 68 | 53 | 22 | 2 | |
| | SRR | 1·00 | 0·91 | 0·75 | 0·71 | 0·65 | 2·80 | N.S. |
| | P | 69 | 14 | 57 | 72 | 53 | 20 | |
| 60–69 | C | 50 | 19 | 77 | 77 | 44 | 18 | |
| | SRR | 1·00 | 0·53 | 0·54* | 0·68 | 0·87 | 0·81 | N.S. |
| | P | 113 | 13 | 87 | 65 | 45 | 32 | |
| 70+ | C | 99 | 18 | 95 | 85 | 40 | 18 | |
| | SRR | 1·00 | 0·63 | 0·80 | 0·67 | 0·99 | 1·56 | N.S. |
| All ages | P | 245 | 73 | 266 | 223 | 130 | 64 | |
| | C | 194 | 82 | 306 | 257 | 120 | 42 | |
| | SRR | 1·00 | 0·70 | 0·69** | 0·69** | 0·86 | 1·21 | < 0·05 |

* $P < 0{\cdot}05$, ** $P < 0{\cdot}01$. Summary chi² test for linear trend with parity: $P < 0{\cdot}001$ ($chi^2 = 19{\cdot}04$).

TABLE VI.—*Estimates of standardized relative risks (SRR) relative to women with their first birth before 20, according to age at first birth after stratification for parity*

| | | Age at first birth (years) | | | | |
|---|---|---|---|---|---|---|
| Parity | | 20 | 20–24 | 24–29 | 30–34 | 35+ |
| 1 | P | 11 | 63 | 56 | 56 | 46 |
| | C | 6 | 60 | 63 | 45 | 22 |
| | SRR | 1·00 | 0·57 | 0·48 | 0·68 | 1·14 |
| 2 | P | 24 | 98 | 78 | 53 | 14 |
| | C | 20 | 99 | 90 | 43 | 13 |
| | SRR | 1·00 | 0·82 | 0·72 | 1·03 | 0·90 |
| 3 | P | 17 | 49 | 49 | 12 | 3 |
| | C | 28 | 75 | 55 | 20 | 6 |
| | SRR | 1·00 | 1·00 | 1·47 | 0·99 | 0·82 |
| 4 | P | 11 | 24 | 26 | 7 | 1 |
| | C | 14 | 32 | 26 | 7 | 1 |
| | SRR | 1·00 | 0·95 | 1·27 | 1·27 | 1·2 |
| 5 | P | 10 | 32 | 14 | 2 | 0 |
| | C | 14 | 40 | 23 | 5 | 0 |
| | SRR | 1·00 | 1·12 | 0·85 | 0·56 | — |
| All parous | P | 73 | 266 | 223 | 130 | 64 |
| | C | 82 | 306 | 257 | 120 | 42 |
| | SRR | 1·00 | 0·98 | 0·97 | 1·22 | 1·71 |

No SRR differed significantly from unity.

Test for trend applied to both horizontal and vertical strata revealed $P < 0{\cdot}05$ for trend with parity for the stratum age at first birth 35+ years. All others were non-significant.

The summary $\chi^2$ for the total material with respect to trend with parity was 7·32 ($P < 0{\cdot}01$) and to trend with age at first birth 2·15 ($P > 0{\cdot}05$).

been real, or due to chance. It also seemed possible that this finding could have been caused by confounding due to a comparatively high parity in this group. The latter possibility was supported by the findings after stratification for parity (Table VI). Trend analysis of the data in Table VI revealed no significant trend with age at first birth after stratification for parity (horizontal strata) but a $P < 0{\cdot}05$ trend with parity for the stratum age at first birth 35+ years (vertical stratum). The summary $\chi^2$ for the total material was significant with respect to trend with parity ($P < 0{\cdot}01$) but insignificant with respect to trend with age at first birth ($P > 0{\cdot}05$).

### DISCUSSION

In a previous case-control study comprising 179 breast-cancer patients, we analysed several factors related to the reproductive history (Adami *et al.*, 1978). No single factor studied was found to have significant influence on the risk of breast cancer. This finding was surprising, and particularly unexpected for the two factors parity and age at first birth. Age at first birth has been frequently discussed during the last decade in connection with endocrine hypotheses (MacMahon *et al.*, 1973). It therefore seemed important to analyse a larger material to see whether these two factors were not indeed associated with a substantial risk in Sweden also.

The present study is based on a material of 1001 patients with carefully age-matched, population controls. The material was obtained from a homogeneous Caucasian population in a defined geographic area, representing a considerable part of Sweden. The non-response rate was low in both the patient and the control groups, and was considered too small to be of significance as a source of bias. We therefore consider the results representative of the whole Swedish population.

An inverse relationship between risk of breast cancer and the number of children a woman has borne is one of the most consistent findings in breast-cancer epidemiology. This relationship has been confirmed by some recent authors in various parts of the world (Paymaster & Gangadharan, 1972; Salber *et al.*, 1969; Shapiro *et al.*, 1973; Soini, 1977; Thein-Hlaing & Thein-Maung-Myint, 1978) but negated by others (Sartwell *et al.*, 1977; Sravraky & Emmons, 1974). According to our study, nulliparous women run a higher risk than ever-parous women. The relative risk is quite modest, however (SRR = 1·35), and the question whether it is influenced by nulliparity or parity of > 2 is a matter of interpretation. The trend is quite regular for women with a parity of 3 or less, but our results do not indicate that pregnancies after the third give a further reduction in the risk of breast cancer. This is in accordance with the findings of Shapiro *et al.* (1973). In the present study the risk for women with a parity of 3+ was found to be about 60% of that for nulliparous women, which is in agreement with the report of MacMahon *et al.* (1970*a*).

A more complex picture has developed since MacMahon *et al.* (1970*a*) demonstrated one decade ago that age at first birth is an important risk factor in different parts of the world. The findings in this international, collaborative investigation, in which hospitalized women were used as controls, were subsequently confirmed in the U.S. by Henderson *et al.* (1974) in a study with outpatients as controls, and also in a community-wide study by Lilienfeld *et al.* (1975). Further support emerged from an analysis of women included in a large screening investigation in Greater New York (the HIP study) (Shapiro *et al.*, 1973) and also from a screening project in Finland (Soini, 1977) as well as from a prospective study set up in Guernsey (Farewell *et al.*, 1977). With hospitalized patients as controls, Wynder *et al.* (1978) found in a large material a protective effect of early age at first birth. This was restricted, however, to pre- and peri-menopausal women. On the other hand, Stavraky & Emmons (1974) found the same influence in postmenopausal women only. One study in the U.S. (Sartwell *et al.*, 1977) and one in Burma (Thein-Hlaing & Thein-Maung-Myint, 1978) showed no significant relationship.

In our material, the analysis of age at first birth as a risk factor did not disclose any clear-cut picture. The risk was lower for those who had first birth before the age of 35 years than for nulliparous women, but in women below 30 years of age at first birth the relative risk was the same in all age groups. Moreover, stratification indicated that the results might be confounded by parity. This finding is contradictory to that in the collaborative study (MacMahon *et al.*, 1970*a*) where the association with parity could be fully explained by the correlation of this factor with the age at first birth. The alternative possibility, that parity and age at first birth act independently, was suggested by Soini (1977) and (for women with their first birth between 20 and 29 years) by Shapiro *et al.* (1973).

We have to conclude that the evidence concerning epidemiological characteristics of breast cancer is contradictory. If parity and age at first birth are indeed risk factors, their influence is generally low, and in most studies, as well as in the present one, they give a relative risk that is less than 2-fold. It therefore seems clear that they cannot account for the large international differences in the incidence of breast cancer (MacMahon *et al.*, 1973; Wynder *et al.*, 1978). Furthermore they cannot be used to define a target population which includes many new cases but is of manageable dimensions, thus enabling regular screening to be carried out for early diagnosis (Farewell *et al.*, 1977; Shapiro *et al.*, 1973).

A question of major importance, then, is whether the relation is causal and can serve as a basis for aetiological hypotheses. We found no clear trend with age at first birth and a low degree of correlation, and observed that stratification for parity eliminated the decrease in relative risk for women with an early first birth. There is also a lack of uniform support from other studies. We therefore rather believe that age at first birth (and perhaps also parity) are associated with other factors of aetiological importance that are yet to be identified.

This study was supported by Grant No. 1088-B78-02X from the Swedish Cancer Society.

We are much indebted to Mrs Elisabeth Sandberg for her invaluable technical assistance. Our thanks are also due to the staff of all participating departments of pathology, whose enthusiastic cooperation greatly facilitated the study.

### REFERENCES

ADAMI, H. O., RIMSTEN, Å., STENKVIST, B. & VEGELIUS, J. (1978) Reproductive history and risk of breast cancer. A case-control study. *Cancer*, **41**, 747.

ADAMI, H. O. & VEGELIUS, J. (1978) A method for estimating bias introduced into epidemiological investigations by those who refuse to participate. *Ann. Clin. Res.*, **10**, 38.

CRAIG, T. J., COMSTOCK, G. W. & GEISER, P. V. (1974) Epidemiologic comparison of breast cancer patients with early and late onset of malignancy and general population controls. *J. Natl Cancer Inst.*, **53**, 1577.

FAREWELL, V. T., MATH, B. & MATH, M. (1977) The combined effect of breast cancer risk factors. *Cancer*, **40**, 931.

HENDERSON, B. E., POWELL, D., ROSARIO, I. & 6 others (1974) An epidemiologic study of breast cancer. *J. Natl Cancer Inst.*, **53**, 609.

LILIENFELD, A. M., COOMBS, J., BROSS, I. D. J. & CHAMBERLAIN, A. (1975) Marital and reproductive experience in a community-wide epidemiological study of breast cancer. *Johns Hopkins Med. J.*, **136**, 157.

MACMAHON, B., COLE, P., LIN, T. M. & 6 others (1970*a*) Age at first birth and breast cancer risk. *Bull. WHO*, **43**, 209.

MACMAHON, B., LIN, T. M., LOWE, C. R. & 6 others (1970*b*) Lactation and cancer of the breast. A summary of an international study. *Bull. WHO*, **42**, 185.

MACMAHON, B. & PUGH, T. F. (1970) *Epidemiology. Principles and Methods.* Boston: Little, Brown & Co.

MACMAHON, B., COLE, P. & BROWN, J. (1973) Etiology of human breast cancer—A review. *J. Natl Cancer Inst.*, **50**, 21.

MANTEL, N. & HAENSZEL, W. (1959) Statistical aspects of the analysis of data from retrospective studies of disease. *J. Natl Cancer Inst.*, **22**, 719.

MANTEL, N. (1963) Chi-square tests with one degree of freedom: Extensions of the Mantel–Haenszel procedure. *J. Am. Stat. Ass.*, **58**, 690.

MIETTINEN, O. S. (1972) Standardization of risk ratios. *Am. J. Epidemiol.*, **96**, 383.

MIETTINEN, O. S. (1976) Estimability and estimation in case-referent studies. *Am. J. Epidemiol.*, **103**, 226.

PAYMASTER, J. C. & GANGADHARAN, P. (1972) Epidemiology of breast cancer in India. *J. Natl Cancer Inst.*, **48**, 1021.

SALBER, E., TRICHOPOULOS, D. & MACMAHON, B. (1969) Lactation and reproductive histories of breast cancer patients in Boston, 1965–66. *J. Natl Cancer Inst.*, **43**, 1013.

SARTWELL, P. E., ARTHES, F. G. & TONASCIA, J. A. (1977) Exogenous hormones, reproductive history, and breast cancer. *J. Natl Cancer Inst.*, **59**, 1589.

SHAPIRO, S., GOLDBERG, J., VENET, L. & STRAX, P. (1973) Risk factors in breast cancer—A prespective study. In *Host Environment Interactions in the Etiology of Cancer in Man.* (Eds Doll & Vodopija). Lyon: I.A.R.C. p. 169.

SOINI, I. (1977) Risk factors of breast cancer in Finland. *Int. J. Epidemiol.*, **6**, 365.

STAVRAKY, K. & EMMONS, S. (1974) Breast cancer in premenopausal and postmenopausal women. *J. Natl Cancer Inst.*, **53**, 647.

SWEDISH CANCER REGISTRY (1979) *Cancer Incidence in Sweden* 1973. Stockholm: National Board of Health and Welfare.

THEIN-HLAING & THEIN-MAUNG-MYINT (1978) Risk factors of breast cancer in Burma. *Int. J. Cancer*, **21**, 432.

WYNDER, E. L., MACCORNACK, F. A. & STELLMAN, S. D. (1978) Epidemiology of breast cancer in 785 United States Caucasian women. *Cancer*, **41**, 2341.